# Reopening schools after the COVID-19 lockdown

**DOI:** 10.7189/jogh.10.010376

**Published:** 2020-06

**Authors:** Aziz Sheikh, Asiyah Sheikh, Zakariya Sheikh, Sangeeta Dhami

**Affiliations:** 1 Usher Institute, University of Edinburgh, Edinburgh, UK; 2 Medical School, University of Edinburgh, Edinburgh, UK; 3 General Practitioner Locum, NHS Lothian, Edinburgh, UK

With nationwide school closures currently operating in 191 countries, the United Nations Educational, Scientific and Cultural Organization (UNESCO) has estimated that 1.6 billion (90.2%) students are currently out of primary, secondary and tertiary education (henceforth schools) as a result of the global COVID-19 lockdown [1]. These restrictions have been introduced to help maintain physical distancing and have contributed to the stabilising incidence of SARS-CoV-2 infections and resulting COVID-19 hospitalizations and deaths now being witnessed in many parts of the world. These measures have the potential however – particularly if prolonged – to result in major detrimental effects on the health and well-being of children and adolescents. In the absence of a robust evidence base on lockdown exit strategies, we consider the range of options being taken globally to reopen schools with a view to informing the formulation of national plans.

It is now well recognized that children and young people can be asymptomatic carriers of SARS-CoV-2 or develop COVID-19 [2]. Although COVID-19 tends to be less severe in children and adolescents, and thankfully relatively few students have died of the condition, a key policy concern has been that young people may be important community reservoirs for the transmission of the virus to household members. Emerging evidence however suggests that children are not super-spreaders of the virus and in fact may not be significantly contributing to spreading the virus [3-5]. A recent (unpublished) systematic review concluded that children and young people under 20 are 56% less likely to contract SARS-COV2 from infected individuals than adults this suggesting they may play a smaller part in transmission than originally thought [6]. It appears therefore that SARS-CoV-2 behaves differently in this respect from many other viral respiratory infections that are responsible for upper respiratory tract infection (URTI) and influenza. A recent systematic review on school closures, which drew primarily on the evidence base from severe acute respiratory syndrome (SARS), concluded that around 2–4% of COVID-19 deaths could be prevented as a result of school closures [7].

Although an important public health intervention in the context of epidemics/pandemics, school closures can have adverse effects on children and adolescents in multiple ways [8]. Not only are they missing out on their education – with potential lifelong implications – children from deprived backgrounds are at increased risk of hunger from missing free school meals, domestic violence, and the poverty that ensues from parents being unable to work because of daytime caring responsibilities. These consequences are felt most by the most vulnerable members of society. The longer lockdowns continue, the greater the risks to the well-being of young people.

What then are the options for reopening schools? The key consideration is how to enable the safe return of as many learners and staff as possible whilst maintaining physical distancing. **Table 1** summarises the approaches that are being employed internationally. We briefly consider these four broad approaches in turn.

**Table 1:** Strategies being adopted internationally to reopen schools after the COVID-19 lockdown

Strategy	Countries
**Maintain closures indefinitely until a vaccine or treatment available**	Current default position for most countries eg, Canada, Israel, Italy, Malta, Spain, UAE, many US states
**Open completely**	Some regions of Japan; Taiwan
**Partial reopening:**	
**By school-level (eg, primary schools)**	Denmark, France, Germany, Iceland, Israel, Mexico, Netherlands, New Zealand, Norway, South Africa, Sweden, Vietnam; regions of China
**Shifts**	Vietnam
**Outdoor schooling**	Denmark
**Hybrid physical and virtual school**	New Zealand, Vietnam; regions of Russia

The first is to maintain school closures until a vaccine can be administered at sufficient levels to achieve herd immunity or a treatment is found. Optimistic estimates suggest that it will be at least 12-18 months before a vaccine is developed and deployed [9]. Given the substantial negative effects of school closures, it seems most unlikely that this will be a tenable strategy for most countries in the medium- to longer-term.

A second approach is to reopen schools completely once the effective reproduction number (R_t_) is well below 1. Whilst this has the benefits of resuming normal schooling, it runs the risk of triggering further peaks in infection. The magnitude of this risk will become clearer as the epidemiology of SARS-CoV-2 transmission in young people becomes better understood. The approach being employed in Denmark whereby children are being taught outdoors and maintaining 2m physical distancing through for example the rearrangement of desks, in an attempt to reduce droplet and contact transmission, could potentially be replicated in a number of other countries [10].

**Figure:**
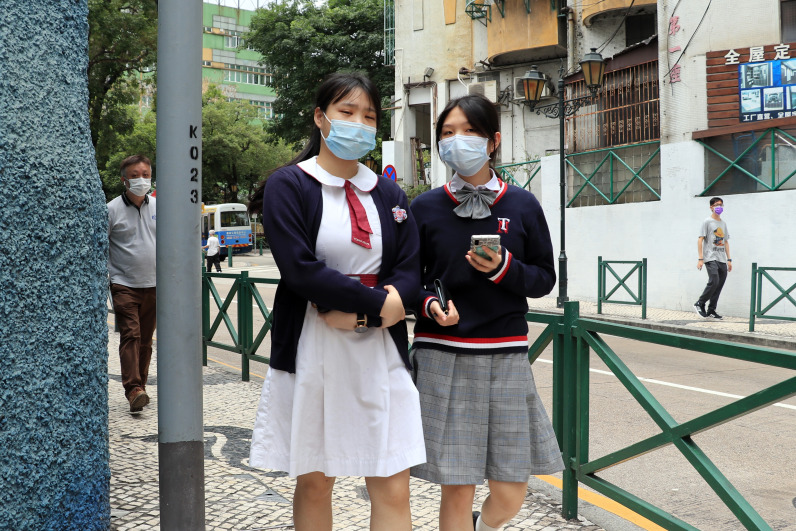
Photo: Macau students return to school. By Macau Photo Agency via Unsplash.

The third strategy is to partially reopen schools such that there are fewer students at school at any one point in time thereby enabling physical distancing. This has been the most popular school lockdown exit strategy employed thus far with students typically attending for part of the week or in shifts.

Finally, a hybrid approach whereby in-person classes are live-streamed to those who for example need to be shielded because of underlying chronic disease or have the capacity to study from home. This is however clearly dependent both on having high speed Internet access and appropriate devices (personal computer, laptop or tablet) at home.

The final three options all need to be accompanied by developing surveillance capability and the ability to rapidly test, trace and isolate suspected COVID-19 cases and their contacts. These also requires capacity for regular deep cleaning of schools to minimise the risk of contact transmission.

It is clear that there are no easy answers. Whichever approach countries choose to take, it is crucial that there are carefully planned evaluations of the approaches employed to help develop a robust evidence base to guide decision making for this and future pandemics.
